# Lapachol inhibits glycolysis in cancer cells by targeting pyruvate kinase M2

**DOI:** 10.1371/journal.pone.0191419

**Published:** 2018-02-02

**Authors:** Mani Shankar Babu, Sailendra Mahanta, Alexander J. Lakhter, Takashi Hato, Subhankar Paul, Samisubbu R. Naidu

**Affiliations:** 1 Department of Microbiology and Immunology, Indiana University School of Medicine, Indianapolis, Indiana, United States of America; 2 Structural Biology and Nanomedicine Laboratory, Department of Biotechnology and Medical Engineering, National Institute of Technology, Rourkela, Odisha, India; 3 Department of Medicine, Indiana University School of Medicine, Indianapolis, Indiana, United States of America; Southern Illinois University School of Medicine, UNITED STATES

## Abstract

Reliance on aerobic glycolysis is one of the hallmarks of cancer. Although pyruvate kinase M2 (PKM2) is a key mediator of glycolysis in cancer cells, lack of selective agents that target PKM2 remains a challenge in exploiting metabolic pathways for cancer therapy. We report that unlike its structural analog shikonin, a known inhibitor of PKM2, lapachol failed to induce non-apoptotic cell death ferroxitosis in hypoxia. However, melanoma cells treated with lapachol showed a dose-dependent inhibition of glycolysis and a corresponding increase in oxygen consumption. Accordingly, in silico studies revealed a high affinity-binding pocket for lapachol on PKM2 structure. Lapachol inhibited PKM2 activity of purified enzyme as well as in melanoma cell extracts. Blockade of glycolysis by lapachol in melanoma cells led to decreased ATP levels and inhibition of cell proliferation. Furthermore, perturbation of glycolysis in melanoma cells with lapachol sensitized cells to mitochondrial protonophore and promoted apoptosis. These results present lapachol as an inhibitor of PKM2 to interrogate metabolic plasticity in tumor cells.

## Introduction

Energy production in normal cells involves breakdown of glucose in the cytoplasm by glycolysis, and subsequent transport of pyruvate into the mitochondria for extraction of electron by oxidative phosphorylation. However, malignant cells reprogram metabolism to avoid toxic radical formation from the electron transport chain of the mitochondria [1]. Tumor cells metabolize glucose even in the presence of oxygen by a process commonly referred to as aerobic glycolysis or the Warburg effect [2]. Apparently, conversion of glucose to pyruvate in aerobic glycolysis yields two ATP molecules, yet there is a general consensus that most of these glycolytic intermediates are diverted to synthesis of macromolecules [3]. Pyruvate produced in aerobic glycolysis is rapidly converted to lactate to regenerate NAD that drives glycolytic reaction forward [4]. This lactate production partly explains the reason for subdued mitochondrial functions in cancer cells, as mitochondrion is a suitable location for NAD regeneration. Another mechanism that ensures prevention of pyruvate entry into mitochondria is silencing of mitochondrial pyruvate transporters in malignant cells [5–7]. Despite these findings of metabolic reprograming in malignant cells, lack of pharmacological tools that specifically target aerobic glycolysis has limited our efforts in exploiting critical metabolic vulnerabilities towards devising effective cancer treatment strategies.

The pyruvate kinase (PK) locus, a key regulator of glycolysis, codes for multiple isoforms. The oncofetal isoform of pyruvate kinase M2 (PKM2) differs from PKM1 by 22 amino acids resulting from alternate splicing [8]. Although normal cells express the PKM1 isoform, fetal tissues and tumor cells predominantly express the PKM2 isoform[8–10], which is enzymatically less active than PKM1. It is generally thought that the less active PKM2 allows accumulation of glycolytic intermediates that meet the macromolecular biosynthetic demands of highly proliferating tumor cells. These metabolic aspects of PKM2 propelled interest in understanding the regulation of its activity in cancer cells. A high throughput screen identified a benzoic acid derivative as a specific inhibitor of PKM2, yet a high concentration of this compound was required to achieve PKM2 inhibition in cells [11]. In a biochemical approach, PKM2 was identified as a target for a potent anticancer agent shikonin [12]. Although shikonin is commonly used as PKM2 inhibitor [12–15], the redox cycling activity of this compound targets mitochondria and limits its use in understanding the role of PKM2 in cancer metabolism [16]. We previously showed that unlike its naphthoquinone analog menadione, shikonin targets both PKM2 and mitochondria in activation of a non-apoptotic cell death termed as ferroxitosis in cells cultured under hypoxia [17]. Despite renewed interest in the role of PKM2 in cancer metabolism, lack of small molecule inhibitors that effectively target PKM2, but not mitochondria, has posed constrain in elucidating the contribution of PKM2 to overall cancer metabolism.

Lapachol has been widely used in traditional medicine to treat various illnesses including cancer [18–23]. The number of patent applications pertaining to anticancer activity of lapachol filed over the past two decades highlights the potential use of this compound as an anticancer agent [24]. Pharmacological studies of lapachol on pregnant rats showed that lapachol was not toxic to mothers but was toxic to the fetus [25]. Due to the fetotoxic effects, potential use of lapachol in cancer research was not explored. Because PKM2 is expressed in fetal tissues and in malignant cells [8, 26], we hypothesized that lapachol may target PKM2. An important clue for assessing the effects of lapachol on pigment producing melanoma cells came from a zebrafish study. A pharmacological study using quinone analogs revealed that lapachol inhibits pigment formation in zebrafish embryos [27]. Despites these clues as potential therapeutics, molecular targets or mechanism of action of lapachol remain to be elucidated. Here we present biochemical, metabolic and computational modeling evidence suggesting that lapachol targets PKM2 in inhibition of glycolysis, and sensitizes melanoma cells to apoptosis.

## Materials and methods

### Cell culture

MEL526, and MEL697 melanoma cell lines were maintained in RPMI1640 (HyClone) medium and MEL103 and A375 cell lines were maintained in DMEM (Life Technologies), supplemented with 10% fetal bovine serum (Sigma), 50 units ml^−1^ penicillin and 50 μg ml^−1^ streptomycin (Life Technologies). Cells were routinely tested for mycoplasma contamination with QuickTest Mycoplasma Detection Kit (Biotool).

### Cell based assays for viability and ATP concentrations

Cells were seeded at density of 1 × 10^4^ cells per well of a 96 well plate (Greiner) in triplicates per condition. Viability was assayed using resazurin reagent (Biotium) and ATP was measured by Cell Titer-Glo 2 assay reagent (Promega) in accordance with manufacturer's protocol. Fluorescence and luminescence signals were read on Synergy H1 microplate reader (BioTek Instruments).

### Cell proliferation

Cells were seeded at a density of 2X10^4^ in triplicates in a 6 well cell culture plate in 5 ml medium and treated with different concentrations of lapachol for a period of 72hours. The culture medium was aspirated and cells were rinsed with phosphate buffered saline and scraped in 1 ml volume of basal DMEM in the absence of fetal bovine serum. The cell suspension was thoroughly mixed and subjected to cell counting in Vi-Cell XR cell counter (Beckman Coulter) using the appropriate program settings in the system. Proliferation rate was calculated as population doublings per day.

### Cellular respirometry

Extra cellular acidification rate (ECAR) and Oxygen consumption rate (OCR) were measured with Seahorse XF96 extracellular flux analyzer (Seahorse Bioscience). Cells were seeded at 1 x 10^4^ cells per well in an XF96 (V3) polystyrene cell culture plate one day before the experiment in the presence of lapachol [sigma 142905] (5 μM, 10 μM, 20 μM, 40 μM) or in the absence of lapachol (0 μM). The ECAR experiments were performed at 37°C in a bath solution consisting of XF assay medium in the absence of glucose supplemented with 2mM glutamine. Each measurement cycle consisted of a mixing time of 1 minute, waiting time for 2 minutes and data acquisition period of 6 minutes. In a typical experiment 3 baseline measurements were taken prior to the addition of any compound and 3 response measurements were taken followed by the sequential injection of compounds glucose 10 mM (Sigma G-5146), oligomycin A 10 μM (Sigma 75351) and Carbonyl cyanide 4-(trifluoromethoxy)phenylhydrazone FCCP 30 μM (Sigma C2920). Once injected, each compound was present in the bath medium for the duration of the experiment. The OCR experiments were performed in basal RPMI 1640 (Lonza 12-702F) medium containing L-glutamine at 37°C. The measurement parameters were the same as for ECAR except that the sequence of compounds were oligomycin A 10 μM (Sigma 75351), FCCP 30 μM (Sigma C2920) and Rotenone 100 μM (Sigma R8875).

### Pyruvate kinase enzyme purification

Human PKM1 (#44241) and PKM2 (#44242) in pET28a vector were purchased from addgene expressed as an N-terminal His_6_ tag fusion protein. pET28a-PKM1 and pET28a-PKM2 was transformed into BL2 (DE3) pLysS cells and grown to an absorbance of 0.8 at 600 nm, then induced with 0.5 mM IPTG for 7 h at room temperature. Cells were lysed in lysis buffer 50 mM Tris pH 8.0, 100 mM NaCl, 1% NP40, 10 mM imidazole, 1 mM PMSF, 1mM DTT and cell lysate was cleared by centrifugation. PKM2 was purified by batch binding to Ni-NTA resin (Qiagen). The resin was then washed with lysis buffer containing 25 mM imidazole for 200 column volumes and His_6_-tag-PKM1 and His_6_-tag-PKM2 was eluted with 200 mM imidazole. The protein was dialysed overnight at 4°C to remove the imidazole in a buffer containing 10mM Tris pH7.4, 100mM NaCl, 0.5mM DTT and 10% glycerol using slide-A-lyzer dialysis cassette (Thermo Scientific) of 10K molecular weight cut off and 0.1–0.5 ml capacity. The enzyme fraction was collected using a 0.5 G needle and stored in 20% glycerol containing dialysis buffer at -80°C in deep freezer in aliquots of 50μL.

### Measurement of pyruvate kinase activity

Pyruvate kinase activity was measured by a continuous assay coupled to lactate dehydrogenase (LDH). The assay for PKM activity determination contained recombinant pyruvate kinase (100 ng) or cell lysate (5μg), Tris pH 7.5 (50 mM), KCl (100 mM), ADP (0.6 mM), PEP (0.5 mM), NADH (180μM) and LDH (8 units). The recombinant enzyme was incubated with the different concentrations of lapachol on ice for 30 minutes and then the buffer containing PEP, ADP, NADH and LDH were added and incubated for 3 hours at room temperature. The ATP generated during the conversion of phosphorenolpyruvate (PEP) to pyruvate was measured in triplicates in a reaction volume of 50 μL by Cell Titer-Glo 2 assay reagent (Promega) in accordance with manufacturer's protocol. Pyruvate kinase activity in cell extracts of the melanoma cell line MEL103 pretreated with different concentrations of lapachol (0 μM, 10 μM, 20 μM and 40 μM) for 24 hours and the enzyme activity was assessed using Pyruvate Kinase Activity (Bio Vision, K709-100). The fluorescence was measured at excitation of 535nm and emission at 587nm showing an increase in pyruvate proportional to the activity of the pyruvate kinase enzyme.

### Flow cytometric analysis of apoptosis

MEL 103 cell lines were seeded at a density of 4X10^4^cells/well in triplicates in a 6 well cell culture plate and treated for 24 hours with DMSO (Vehicle), lapachol (20 μM), DNP (10 μM) and lapachol (20 μM) + DNP(10 μM). The cells were then stained for annexin 7AAD and PE analysis using the PE Annexin V apoptosis detection kit (BD Pharmingen 559763) following manufacturer’s instructions. After staining the cells were subjected to flow cytometry analysis in a LSR II flow cytometer (Becton and Dickinson) using the appropriate lasers.

### Confocal live cell imaging of mitochondrial membrane potential

To determine mitochondrial membrane potential, MEL 103 cells cultured on a glass bottomed 24 well culture plate at a density of 2X10^4^ were treated with the appropriate concentrations of the drugs overnight. Before the microscopic analysis the cells were incubated with 5 nM tetramethylrhodamine, methyl ester (TMRM) (Thermo Fisher). Time lapse images were obtained with a custom spinning-disk microscope built around a CSU-10 Confocal head (Yokogawa), a 897 Xion EMCCD camera (Andor), a 4-line monolithic laser launch (Agilent) and a TiE inverted microscope stand (Nikon) equipped with a stage top incubator that regulates CO2 at 5% and temperature at 37°C. The cells were imaged at an interval of 3 minutes for a total period of 3 hours.

### Statistical analyses

Unless stated otherwise, data presented are mean ± SD in triplicates per condition. Statistical analyses were done using GraphPad Prism 5, with statistical significance determined by Holm-Sidak method.

## Results

### Shikonin but not lapachol promotes ferroxitosis in hypoxia

**Fig 1:**
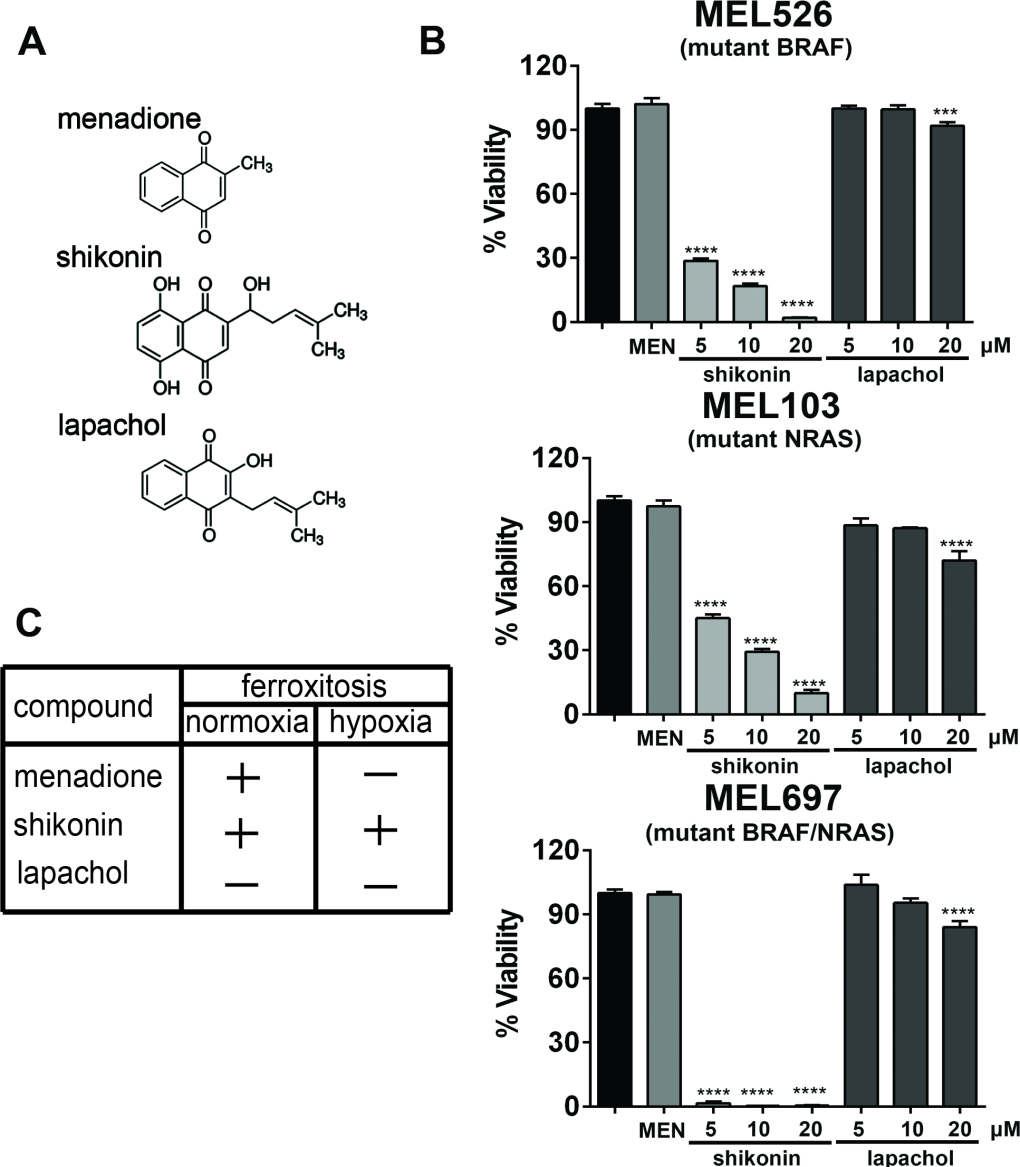
Shikonin but not lapachol promotes ferroxitosis in hypoxia. (A) Chemical structures of naphthoquinone analogs, shikonin [5,8-dihydxroxy-2-(1-hydroxy-4methyl-3-pentenyl)-1,4-naphthoquinone], menadione [2-methyl-1,4-naphthoquinone] and lapachol [2-Hydroxy-3-(-3- methyl-2-butenyl-)-1,4-naphthoquinone]. (B) MEL103 (NRAS^Q61L^), MEL697 (NRAS^Q61L^ BRAF^V600E^) and MEL526 (BRAF^V600E^) were treated with 20μM menadione, 5–20 μM shikonin, or 5–20 μM lapachol for 12 hours in a hypoxic chamber. Viability was determined in a resazurin-based assay. (n = 3/condition) **** P<0.0001. (C) Table depicting the ability of menadione, shikonin and lapachol to induce ferroxitosis under normoxic and hypoxic conditions (“+”—induces ferroxitosis, “_”- does not induce ferroxitosis).

Ferroxitosis is a novel, non-apoptotic cell death dependent on iron and oxygen [17]. We previously reported the mechanism by which napthoquinone analogues menadione and shikonin induce robust mitochondrial oxygen consumption and promote iron- and oxygen-dependent cell death termed ferroxitosis in normoxia [17]. In hypoxia, activation of ferroxitosis by menadione was contingent upon depletion of PKM2. Consistent with these observations, menadione analog and a PKM2 inhibitor, shikonin promoted ferroxitosis in hypoxia [17]. Because lapachol is an analog of shikonin (Fig 1A), we tested the biological effects of this compound on cells under hypoxia. To evaluate the cytotoxic effects of naphthoquinones, MEL526, MEL103 and MEL697 cells under hypoxia were treated with menadione (20 uM), shikonin (5, 10 and 20uM) or lapachol (5, 10 and 20uM), and cell viability was determined (Fig 1B). As we reported, menadione failed to promote cell death in hypoxia and shikonin induced ferroxitosis in a dose-dependent manner in all the cell lines tested. However, lapachol showed a moderate decrease in cell viability at high concentrations. Despite the structural similarities to shikonin, lapachol failed to promote ferroxitosis in hypoxia (Fig 1C). Furthermore, cell viability determination in normoxia also revealed that unlike menadione, lapachol was not cytotoxic to cells (S1 Fig).

### Lapachol inhibits glycolysis in melanoma cells

**Fig 2:**
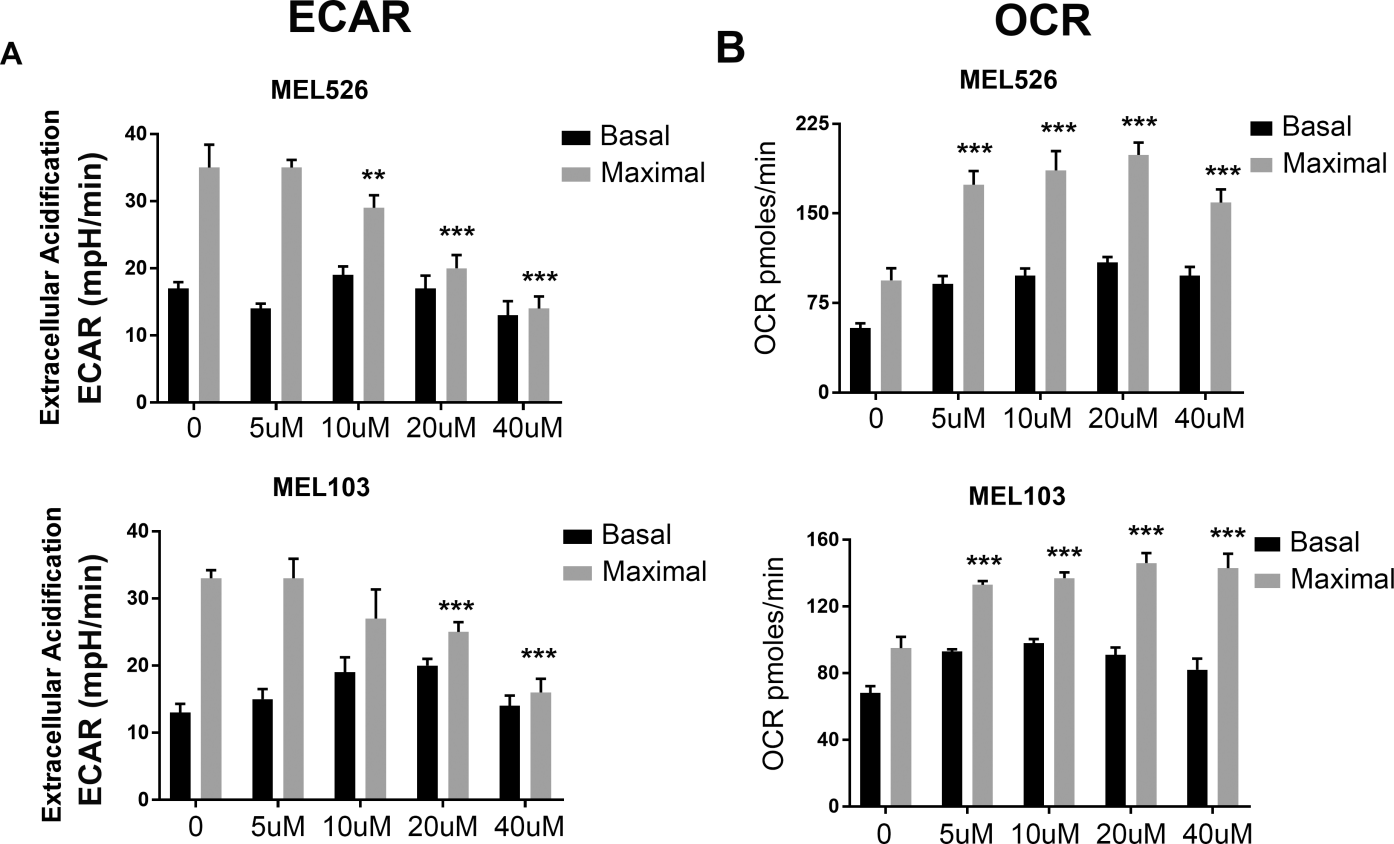
Lapachol inhibits glycolysis in melanoma cells. (A) Extracellular acidification rate (ECAR) under basal (glycolysis) and maximal (glycolytic capacity) after the addition of oligomycin A (10 μM) were plotted for MEL526 and MEL 103 cell lines after the treatment of cell lines with lapachol at concentrations 0 μM, 5 μM, 10 μM, 20 μM and 40 μM overnight. Each value represents mean ECAR value ± se from five replicates (B) Oxygen consumption rate (OCR) under basal and maximal (after the addition of FCCP 30 μM) were plotted for MEL 526 and MEL103 cell lines after the treatment of cell lines with lapachol at concentrations 0 μM, 5 μM, 10 μM, 20 μM and 40 μM overnight.

Although the anti-cancer activity of lapachol has been well recognized [18, 20, 22, 28], our results showed that the cytotoxic effects of lapachol were markedly different from its structural analog shikonin. We hypothesized that lapachol inhibits tumor cell glycolysis in exerting anti-cancer activity. To test this possibility, lapachol treated MEL526 and MEL103 cells were subjected to Seahorse analysis. Lapachol showed a dose-dependent decrease in extra cellular acidification rate (ECAR) in both MEL526 and MEL103 cells (Fig 2A). The effects of lapachol on glycolytic capacity were prominent as high concentrations of lapachol completely blunted glycolytic capacity in melanoma cells. To assess if this inhibition of glycolysis increases oxygen consumption, melanoma cells were treated with different concentrations of lapachol and oxygen consumption rate (OCR) was recorded. Consistent with a decrease in lapachol-mediated ECAR, a dose-dependent increase in OCR was observed in cells exposed to lapachol (Fig 2B). These results establish that lapachol inhibits glycolysis as a result of it, cells switch to oxidative phosphorylation for cell survival.

### In silico studies of shikonin and lapachol interactions with PKM2 protein structure

**Fig 3:**
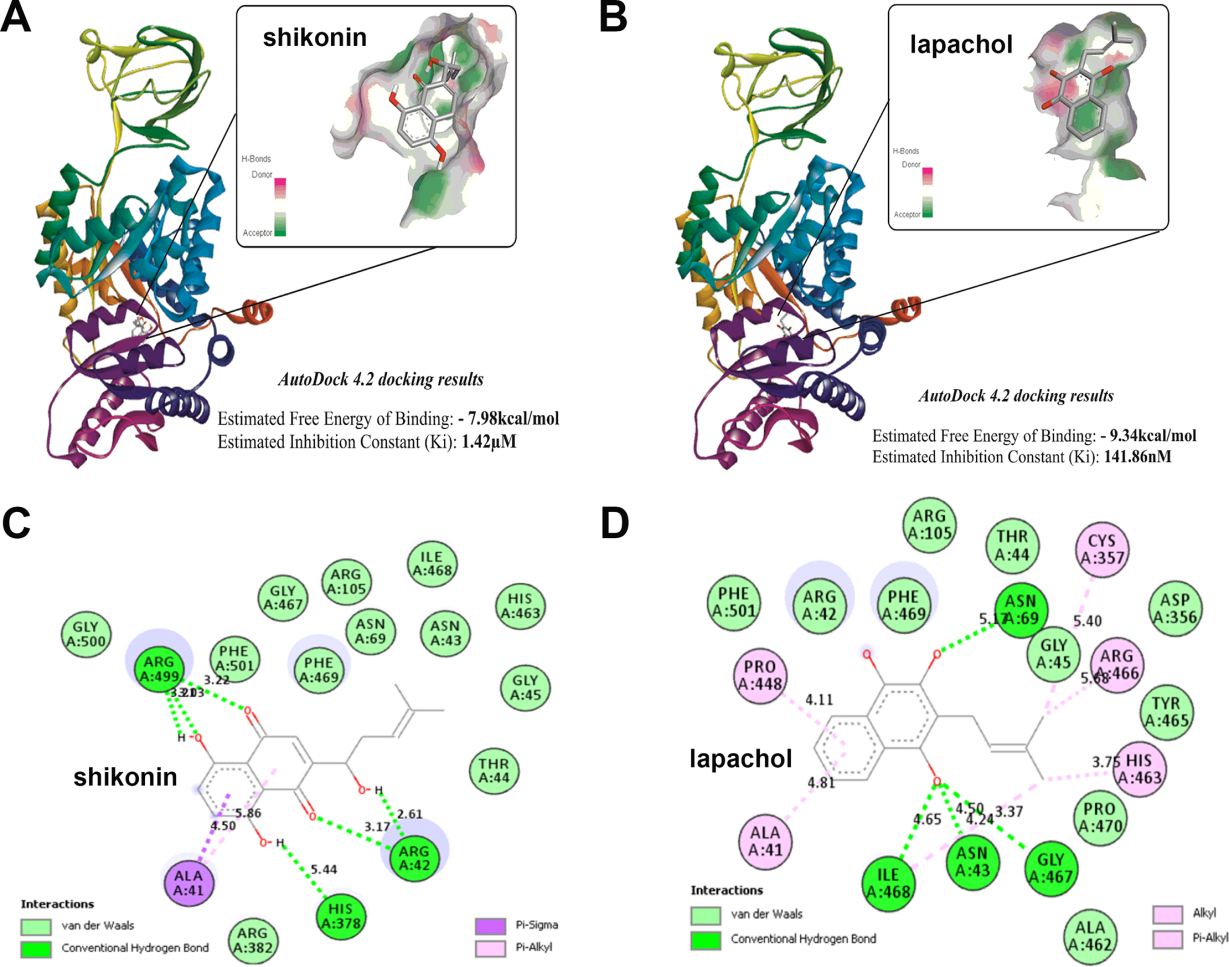
In silico studies of lapachol-PKM2 interactions. (A) The structure of human muscle Pyruvate kinase M2 [1ZJH—Receptor] docked to shikonin (ligand) using Autodock 4.2 software and ligand receptor interaction diagram generated using Discovery studio 2.5. The predicted free energy of binding for shikonin -7.98 kcal/mol and predicted inhibition constant was 1.42 μM. Inset shows the site pocket with surface charge distribution on donor and acceptor atoms. (B) The structure of human Pyruvate kinase M2 docked to lapachol and ligand receptor interaction diagram generated using Discovery studio 2.5. Inset shows the site pocket with surface charge distribution on donor and acceptor atoms. (C) The 17 amino acid residues of human PKM2 protein predicted to interact with the functional groups in shikonin and the type of interaction Van der waals (pale green), H-bond (bright green), Pi-Sigma (purple) and Pi-Alkyl (pink) are highlighted. (D) The 20 amino acid residues of human PKM2 protein predicted to interact with the functional groups in lapachol and the type interaction are highlighted.

Based on our results that lapachol inhibits glycolysis in melanoma cells, we hypothesized that lapachol interacts with the key glycolysis regulator, PKM2. The 3D structure of the PKM2 protein (1ZJH) was obtained from Protein Data Bank (PDB) to identify potential binding pockets for lapachol/shikonin. Energy minimized structures of PKM2, shikonin and lapachol were subjected to molecular docking studies using AutoDock 4.2. Our initial binding site studies using the Lamarckian Genetic Algorithm followed by binding site analysis using Discovery Studio Visualizer Software identified 18 potential binding cavities based on size and volume (S2 Fig). The docking process of AutoDock4.2 was initiated to calculate the binding free energy for shikonin and lapachol on PKM2 macromolecular structure. Fig 3A shows the binding pocket for shikonin (Fig 3A). The estimated free energy of shikonin binding to PKM2 was found to be -7.98 kcal/mol and the inhibition constant was 1.42 μM. Similarly, Fig 3B shows the binding pocket for lapachol (Fig 3B). The estimated free energy of binding of lapachol and inhibition constant were -9.34 kcal/mol and 141.86 nM, respectively. The results show that lapachol has a higher binding affinity for PKM2 compared to shikonin and demonstrates 50% inhibition of PKM2 at a much lower concentration of 141.86 nM. Fig 3A and 3B show that shikonin and Lapachol share a common binding site (Binding Site 2) (Fig 3A and Fig 3B and S3 Fig) on PKM2. However, in order to validate the above findings, we performed a 2D interaction analysis of the binding site using Discovery Studio Visualizer. The 2D interaction analysis revealed the key residues present at the binding site for both the molecules shikonin and lapachol. The results of the 2D interaction analysis of binding site of PKM2 (1ZJH) and the ligand shikonin show the presence of sixteen amino acid residues (Fig 3C). Sixteen amino acid residues, ASN43, GLY45, ASN69, ARG105, GLY467, ILE468, TYR465, PHE501, PHE469, GLY500, THR44, ARG382 show Van der Waal’s interaction with the ligand shikonin. The amino acid residues ARG42, ARG382 and ARG499 share hydrogen bond, and ALA41 has a Pi-alkyl and Sigma alkyl interaction with the ligand shikonin. The results of the 2D interaction analysis of PKM2 (1ZJH) and lapachol show the presence of nineteen residues (Fig 3D). ARG42, GLY45, ARG105, PHE469, PHE501, ALA462, ASP356, TYR465, PRO470 and THR44 show Van der Waal’s interaction with the ligand lapachol. The amino acid residues ASN43, ASN69, GLY467 and ILE468 share a hydrogen bond. ALA41 and PRO448 have a Pi-alkyl interaction with the ligand lapachol. The amino acid residues CYS357, ARJ466 and HIS463 show an alkyl interaction with the ligand lapachol. Shikonin and lapachol share a common nine amino acid interactions and suggest that these two compounds interact with PKM2 at the same binding-pocket.

### Lapachol inhibits PKM2 activity

**Fig 4:**
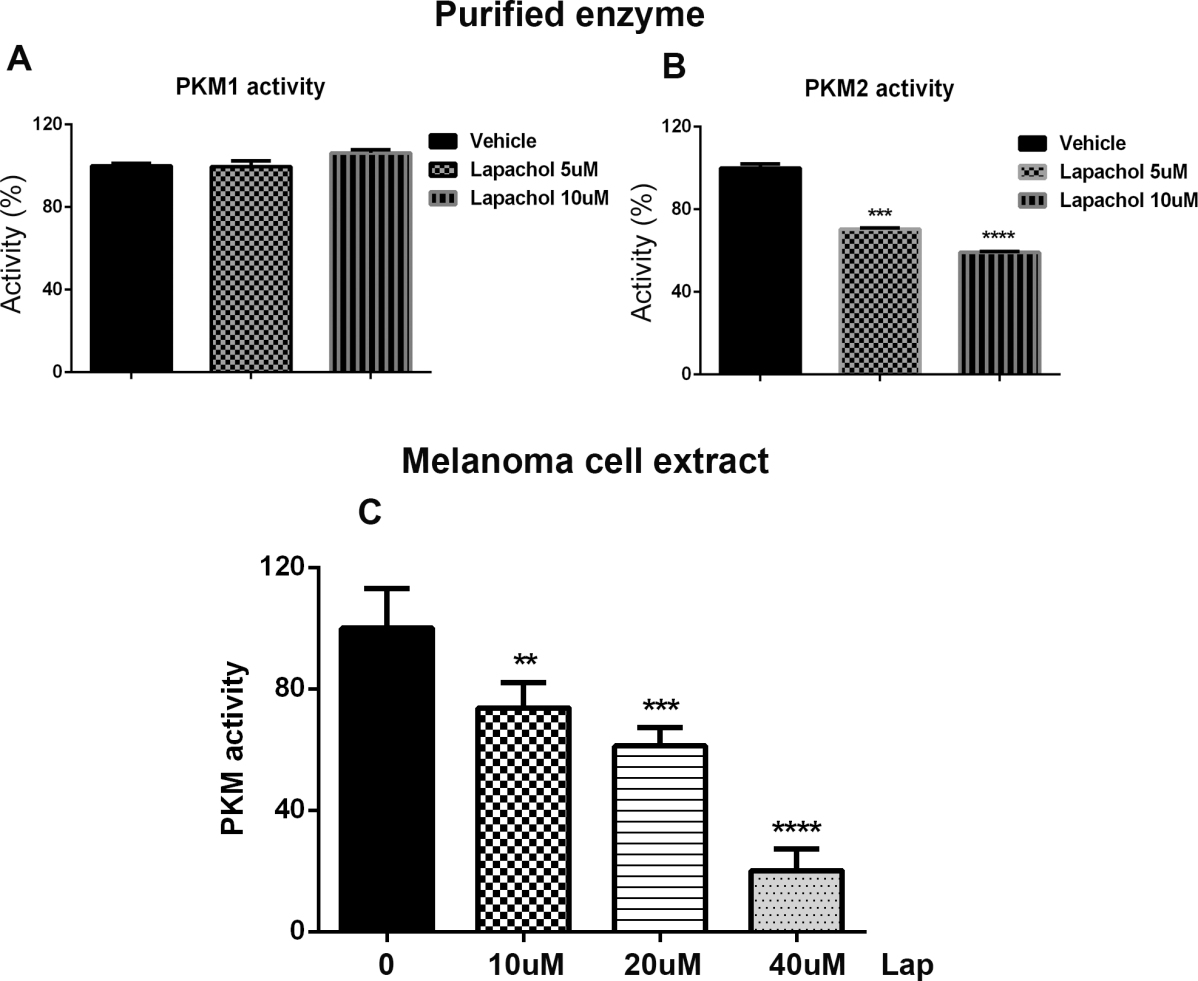
Lapachol inhibits PKM2 enzyme activity. (A) Recombinant human PKM1 enzyme was isolated and in vitro PKM1 enzyme activity was measured using a continuous assay coupled to lactate dehydrogenase. Different concentrations of lapachol (vehicle, 5 μM and 10 μM) did not show an inhibition in the activity of recombinant PKM1 invitro. (B) lapachol inhibited the activity of recombinant PKM2 in a dose dependent manner (n = 3/condition) **** P<0.0001 (C) Lapachol inhibited the pyruvate kinase activity invivo in the melanoma cell line MEL103 in a dose dependent manner (n = 3/condition)****P<0.0001.

Results of our metabolic and computational docking studies led us to test whether lapachol inhibits the enzyme activity of PKM2. Bacterially produced human PKM1 and PKM2 were purified and the enzyme activity was determined in the presence of lapachol. Although 10 uM lapachol inhibited 50% of PKM2 activity, PKM1 activity was not altered by 10 uM lapachol (Fig 4A and 4B). To further validate these results, MEL103 cells were exposed to various concentration of lapachol and the enzyme activity of PKM2 was determined in the cell extracts. Lapachol inhibited PKM2 activity in the cell extracts in a dose dependent manner (Fig 4C).

### Lapachol inhibits melanoma cell proliferation

**Fig 5:**
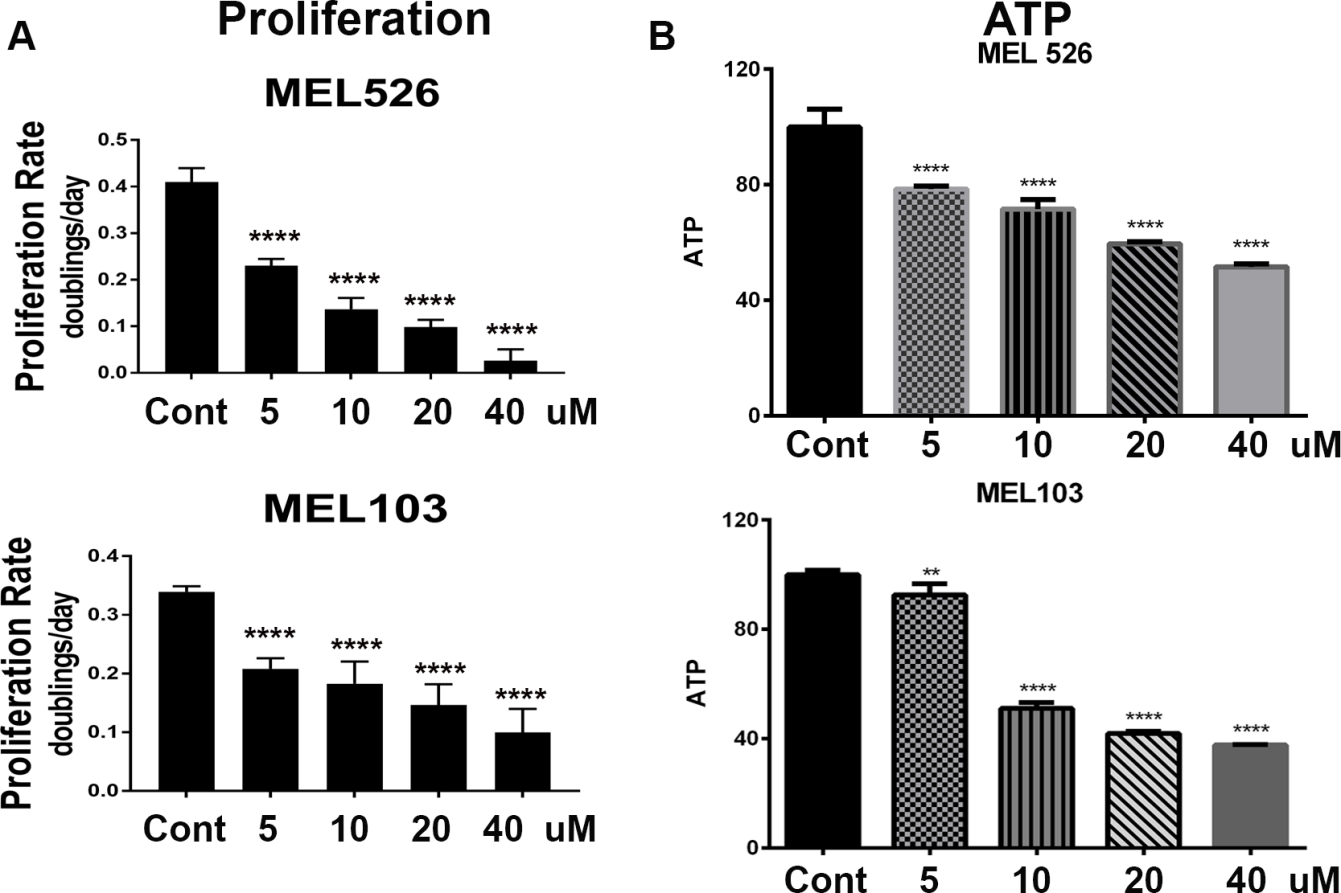
Lapachol inhibits melanoma cell proliferation. (A) Cell proliferation was determined for MEL 526 and MEL103 melanoma cell lines and plot show the rate of proliferation in the presence of increasing concentrations of lapachol (5 μM, 10 μM, 20 μM, 40 μM) (n = 3/condition)****P<0.0001 (B) Cellular ATP levels were measured in MEL 526 and MEL103 melanoma cell lines in the presence of increasing concentrations of lapachol (5 μM, 10 μM, 20 μM, 40 μM) (n = 3/condition)****P<0.0001.

Our results showed that lapachol was not cytotoxic to melanoma cells, yet it inhibited PKM2 activity and glycolysis. Therefore, we tested whether lapachol inhibits cell proliferation. MEL526 and MEL103 cells were exposed to various concentrations of lapachol and cell number was quantified to determine cell proliferation. As much as 10 uM lapachol was sufficient to reduce cell proliferation to half of control cells (Fig 5A). High concentrations of lapachol (20–40 uM) significantly inhibited the rate of proliferation in both MEL526 and MEL103 cells. To ascertain these results, we opted to measure ATP levels after exposure to lapachol. Although MEL526 cells showed a dose-dependent decrease in ATP levels, 10 uM lapachol inhibited 50% of ATP levels in MEL103 cells (Fig 5B). The differential effects of lapachol on ATP levels in these cells could be attributed to differences in oncogenic background, as MEL526 is a BRAF mutant and MEL103 harbors an NRAS mutation.

### Lapachol sensitizes melanoma cells to apoptosis

**Fig 6:**
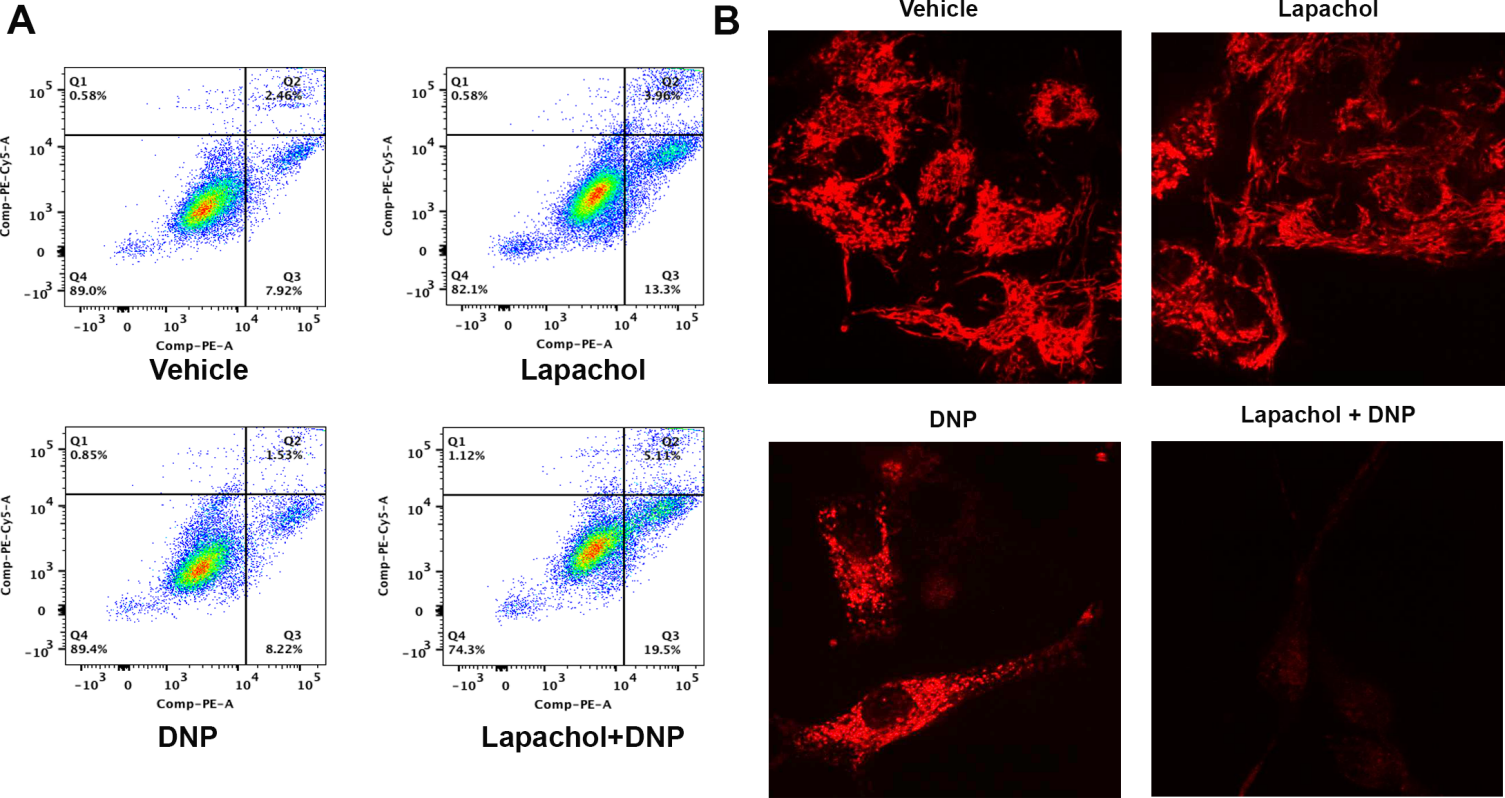
Lapachol sensitizes melanoma cells to apoptosis. (A) Annexin 7AAD-PE analysis of apoptosis carried our in MEL103 cell line. Treatment with DMSO (Vehicle) or 2, 4-di nitro phenol (DNP 10 μM) alone showed low percentage of annexin positive cells 7.9% and 8.2% respectively. Lapachol (20 μM) treatment increased annexin positive cells to 13% but the combined treatment of lapachol (20 μM) and DNP (10 μM) further increased the percentage of annexin positive cells to 20%. (B) Loss of mitochondrial membrane potential was analyzed by TMRM dye in MEL 103 melanoma cell line after treatment with DMSO (Vehicle), lapachol (20 μM), DNP (10 μM) and lapachol (20 μM) +DNP (10 μM). Mitochondrial membrane integrity in vehicle, lapachol (20 μM) and DNP (10 μM) treated cells and loss of mitochondrial membrane potential is revealed in cells treated with a combination of lapachol (20 μM) and DNP (10 μM) as reflected by the loss of TMRM fluorescence intensity.

Metabolic plasticity allows melanoma cells to switch from glycolysis to oxidative phosphorylation in the development of resistance to chemotherapy [29–32]. To test if inhibition of glycolysis with lapachol sensitizes melanoma cells to the mitochondrial drug dinitrophenol (DNP), MEL103 cells were treated with lapachol or DNP or a combination of lapachol and DNP for overnight and apoptosis was assessed by flow cytometry. Although MEL103 cells exposed to lapachol or DNP showed about 10% apoptotic cells, lapachol combined with DNP showed a 20% increase in apoptosis (Fig 6A). To substantiate these observations, we assessed live cell imaging with a mitochondrial dye TMRM, a commonly used method to evaluate mitochondrial membrane potential. MEL103 cells were exposed to lapachol or DNP or combination of these drugs for 12 hours and treated with TMRM prior to live-cell imaging (S1–S4 Videos). MEL103 cells exposed to lapachol or DNP maintained TMRM fluorescence suggesting mitochondrial integrity, whereas the combination of these drugs led to loss of mitochondrial membrane potential (Fig 6B).

## Discussion

The Warburg effect or aerobic glycolysis is a metabolic hallmark of malignancy [2]. Lack of small molecule inhibitors that directly target this metabolic dependency remains a major obstacle in studying cancer metabolism and developing an effective therapy strategy for cancer. Here we present lapachol as an inhibitor of a key glycolytic enzyme, PKM2. Unlike its analog shikonin, lapachol failed to induce ferroxitosis in hypoxia. However, lapachol is an effective inhibitor of glycolysis in melanoma cells. Lapachol inhibit PKM2 activity and reduces ATP levels to inhibit cell proliferation. Furthermore, lapachol-mediated inhibition of glycolysis sensitized melanoma cells to apoptosis. Collectively our findings present lapachol, a novel small molecule that was used in traditional medicine to treat various illnesses for centuries [18–23], as an inhibitor of the glycolytic enzyme PKM2.

Initial studies on lapachol as a potential chemotherapy agent were reported in 1946 [33]. The anti-cancer activity of lapachol when tested against leukemia, sarcoma and Walker 256 carcinoma cells showed that only Walker 256 carcinoma cells were sensitive to this compound [34]. Followed by an oral toxicity study, a clinical study on lapachol was initiated in 1974 to treat malignancy [18]. This study concluded that high concentrations of lapachol were required as a chemotherapy agent, but due to its toxic side-effects, this study was discontinued. In a small study with nine patients with various tumor types (liver, kidney, breast, prostate and cervix), lapachol treatment showed remission in three of the patients. Failures of these studies conducted nearly three decades ago can be explained by recent understanding of cancer metabolism. Despite its critical role in glycolysis, depletion of PKM2 did not significantly reduce tumor growth and deletion of PKM2 did not alter tumor progression in mice [35], suggesting that metabolic plasticity allows tumors to switch to alternate metabolism in sustaining tumor growth. Indeed, our findings that glycolysis inhibition by lapachol sensitized tumor cells to the mitochondrial drug DNP demonstrate that a combination of agents that target both tumor cell glycolysis and mitochondrial metabolism may be an effective strategy to treat cancer. Although lapachol inhibits glycolysis similar to its analog shikonin, the biological outcomes of these analogs differ. We previously showed that in addition to targeting PKM2, shikonin promotes a robust mitochondrial respiration in causing ferroxitosis [17]. Unlike shikonin, lapachol was unable to increase robust oxygen consumption in a manner that causes ferroxitosis [17]. Therefore, lapachol is a novel natural product and can be used as a tool to target tumor cell glycolysis and better understand cancer metabolism.

In general, most chemotherapy approaches target glycolysis in tumor cells [36, 37]. BRAF inhibitors are the first line of therapy for melanoma cases harboring oncogenic BRAF mutation [38, 39]. BRAF inhibition results in reducing glucose uptake and glycolysis [40, 41]. In fact, the activity of BRAF inhibitors on tumor cell metabolism is reminiscent of the effects of lapachol. BRAF inhibitors are selective for mutant BRAF tumors. Because aerobic glycolysis is characteristic of cancer cells, lapachol can be used to block glycolysis regardless of the oncogenic mutations. Our data show that both BRAF and NRAS mutant melanoma cell proliferation is reduced by lapachol. Furthermore, NRAS mutant MEL103 cells treated with lapachol were sensitive to DNP in promoting apoptosis.

It was proposed that PKM2 adopts two major states in regulation of glucose metabolism in cells [42]. A highly active tetrameric and a less active dimeric form were detected in cells. Interestingly, the less active dimeric form of PKM2 was commonly observed in cancer cells and attracting interest in understanding this PKM2 isoform [42]. It is conceivable that cancer cells prefer the dimeric form of PKM2, as rapid conversion of glucose to pyruvate would deplete glycolytic intermediates essential for the synthesis of nucleotides and amino acids. Although shikonin directly binds to PKM2 and has long been used as an inhibitor for PKM2 [12, 13, 43, 44], the binding site for this compound on PKM2 was unknown. Our in silico studies revealed that these napthaquione analogs (shikonin and lapachol) dock to the regulatory domain C in the PKM2 protein structure. We observed a more diverse set of interactions in the case of lapachol with PKM2, which contributes to increased affinity for the binding site (-9.34kcal/mol) compared to shikonin (-7.98kcal/mol). It is important to recognize that the well-known key activator of PKM2, fructose 1,6-biphosphate directly binds to domain C and stimulates the activity of this enzyme [45]. Based on our in silico and biochemical studies, we propose that lapachol binds to this regulatory domain and inhibits PKM2 activity.

Although our findings reveal lapachol as a tool to study cancer metabolism, it is important to address potential clinical implications of presented results. Our data reveal lapachol as an inhibitor of glycolysis, which is critical for tumor cell proliferation. Although lapachol shows inhibitor activity in the micro molar range, the activity of this compound needs to be improved to test in preclinical models. Lapachol docks onto the regulatory domain of PKM2 and provides new structural information that can form a foundation to synthesize derivatives of lapachol with improved activity. There is evidence suggesting that anti-diabetic drug phenformin targets mitochondria and is a powerful anticancer agent [46]. Therefore, an effective anticancer strategy may be to combine improved lapachol derivatives with phenformin to treat cancers regardless of the oncogenic mutations.

## Supporting information

S1 Fig Unlike menadione, lapachol fails to promote ferroxitosis. (PDF)

S2 Fig Potential lapachol binding sites on PKM2. (PDF)

S3 Fig Amino acid residues on PKM2 binding pocket. (PDF)

S1 Video Files1 –Control. (MOV)

S2 Video Files2 –Lapachol. (MOV)

S3 Video Files3 –DNP. (MOV)

S4 Video Files4 –Lapachol+DNP. (MOV)
