# Supplementary material for: Lapachol inhibits glycolysis in cancer cells by targeting pyruvate kinase M2
Source: PLoS One. 2018 Feb 2;13(2):e0191419. doi: 10.1371/journal.pone.0191419 (PMC5796696; doi:10.1371/journal.pone.0191419)
Supplement: S1 Fig — (PDF) [file pone.0191419.s001.pdf]

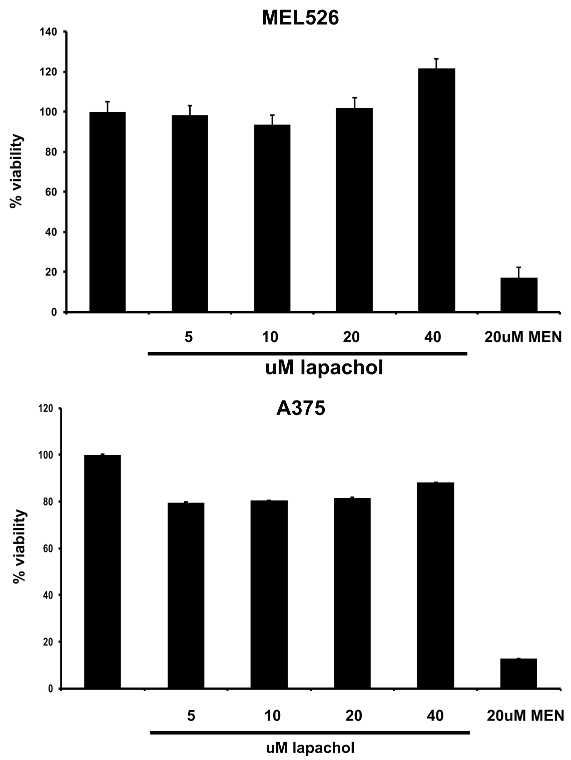

Fig S1. MEL526 or A375 cells were treated with lapachol or 20 uM menadione at 20% oxygen and cell viability shown.
