# Supplementary material for: Lapachol inhibits glycolysis in cancer cells by targeting pyruvate kinase M2
Source: PLoS One. 2018 Feb 2;13(2):e0191419. doi: 10.1371/journal.pone.0191419 (PMC5796696; doi:10.1371/journal.pone.0191419)
Supplement: S2 Fig — (PDF) [file pone.0191419.s002.pdf]

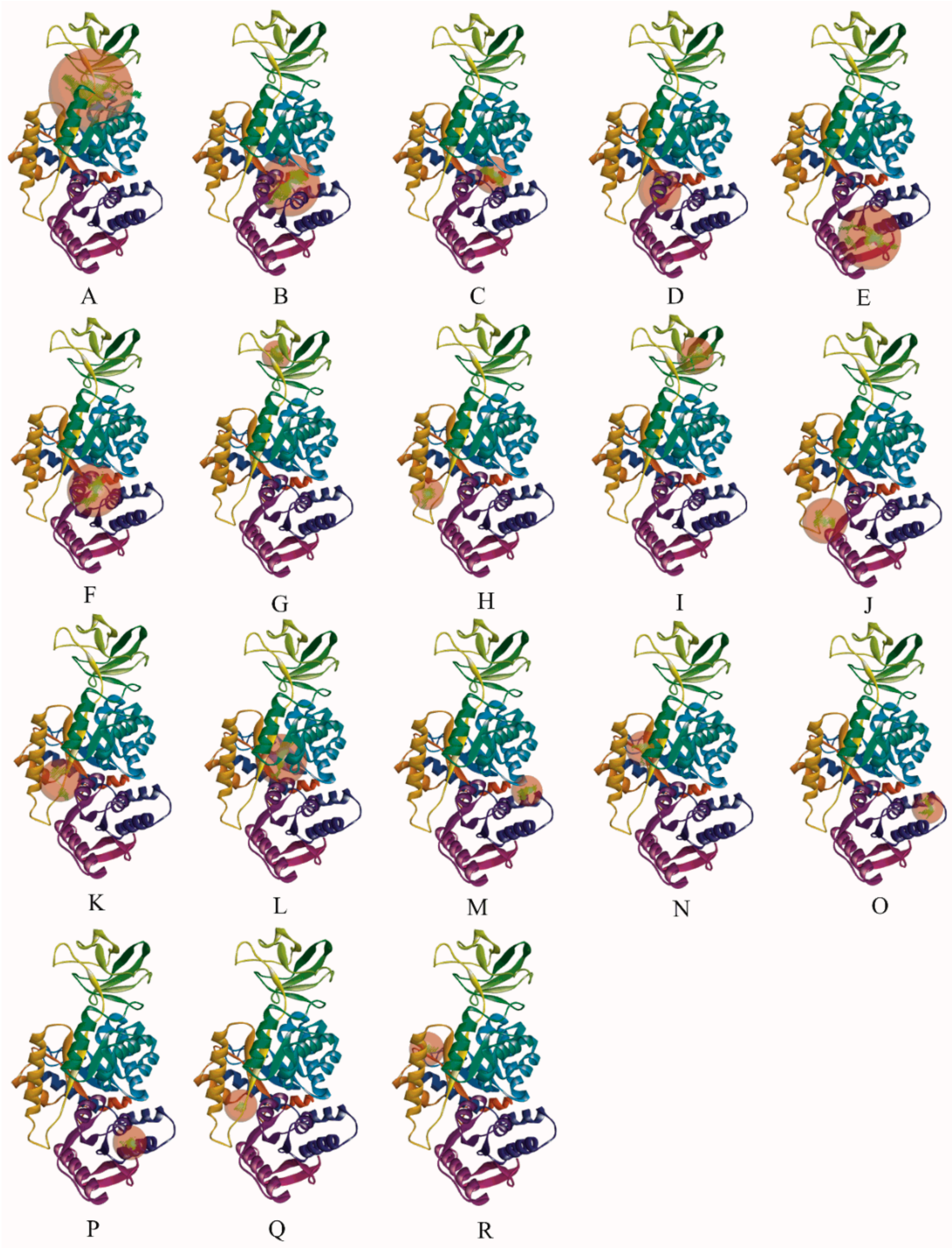

Fig. S2. Binding sites available within the protein PKM2 (1ZJH). The binding sites are surrounded by a red colored sphere for identifying the location within the protein. (A) Site 1 (B) Site 2 (C) Site 3 (D) Site 4 (E) Site 5 (F) Site 6 (G) Site 7 (H) Site 8 (I) Site 9 (J) Site 10 (K) Site 11 (L) Site 12 (M) Site 13 (N) Site 14 (O) Site 15 (P) Site 16 (Q) Site 17 (R) Site 18.
