# Supplementary material for: Lapachol inhibits glycolysis in cancer cells by targeting pyruvate kinase M2
Source: PLoS One. 2018 Feb 2;13(2):e0191419. doi: 10.1371/journal.pone.0191419 (PMC5796696; doi:10.1371/journal.pone.0191419)
Supplement: S3 Fig — (PDF) [file pone.0191419.s003.pdf]

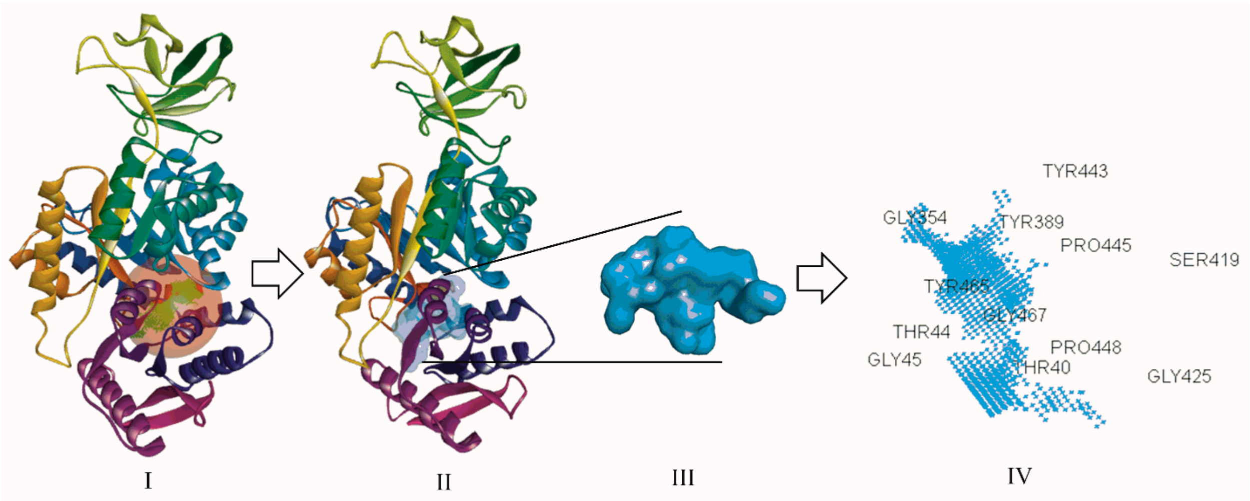

Fig. S3 Identification of the amino acid residues available on the PKM2 receptor-binding site.

Supplemental video files:

1. Control: TMRM imaging of control cells.
2. Lapachol: TMRM imaging of lapachol-treated cells.
3. DNP: TMRM imaging of DNP-treated cells.
4. Lapachol+DNP: TMRM imaging of lapachol+DNP-treated cells.
